# Characterization of Actin Filament Dynamics during Mitosis in Wheat Protoplasts under UV-B Radiation

**DOI:** 10.1038/srep20115

**Published:** 2016-01-29

**Authors:** Huize Chen, Rong Han

**Affiliations:** 1 Higher Education Key Laboratory of Plant Molecular and Environment Stress Response (Shanxi Normal University) in Shanxi Province, Linfen 041004, P. R. China; 2 School of Life Science, Shanxi Normal University, Linfen 041004, P. R. China

## Abstract

Enhanced ultraviolet-B (UV-B) radiation is caused by the thinning ozone and affects photosynthesis and crop yield. Recently, UV-B radiation has been considered as an environmental signal that regulates plant growth. Elucidating the downstream effectors in UV-B-triggered pathways is of particular interest. Previous studies have shown that actin filaments (AFs) play many roles during cell physiological processes. However, the underlying response of AFs to UV-B radiation remains unclear. In this study, wheat protoplasts were isolated from 7-d-old leaves. The dynamics of AFs during mitosis were observed under different treatments. The protoplasts were treated with UV-B radiation, cytochalasin B (CB) and jasplakinolide (JAS). Ph-FITC labelling results revealed typical actin filament structures in the control group; AFs were rearranged under UV-B radiation. AFs polymerized into bundles during interphase, the preprophase band (PPB) structure was destroyed during prophase, and the AFs gathered into plaques during metaphase in response to UV-B radiation. During anaphase and telophase, the distribution of AFs was dispersed. Pharmacologic experiments revealed that CB induced apoptosis and JAS induced nuclear division without cytokinesis in wheat protoplasts. These results indicated that AFs respond to UV-B radiation during mitosis, supplying evidence of UV-B signal transduction in plants.

Ultraviolet (UV) represents almost 7% of the electromagnetic radiation emitted from the sun, which is Earth’s primary energy source. Although 90% of UV-B radiation is absorbed by the stratospheric ozone, the remaining UV-B radiation is transmitted to the Earth’s surface. A 30% increase in UV-B levels would seriously affect crop production in the USA and China^1^. High doses of UV-B radiation can damage nucleic acids and plasma membranes, reduce photosynthesis, and induce the production of reactive oxygen species (ROS)^2^,^3^. However, UV-B radiation was recently found to act as a signalling stimulus that regulates many metabolic processes. The UV-B-specific photoreceptor UV Resistance locus 8 (UVR8) interacts in a strictly UV-B-dependent manner with the ubiquitin ligase Constitutively Photomorphogenic 1 (COP1). These proteins accumulate in the nucleus, where they control gene expression^4^. Additional components of the UVR8-signalling cascade include Elongated Hypocotyl 5 (HY5) and Repressor of UV-B Photomorphogenesis 1 and 2 (RUP1 and RUP2)^5^. Researchers are working to elucidate the mechanisms of UV-B radiation and related signalling pathways in plants.

As widely distributed structures in cells, actin filaments participate in many important cellular processes in plants, including the cell cycle. The arrangements of actin filaments during mitosis in the tobacco cell line Bright Yellow-2 (BY-2)^6^ and wheat root meristematic zone cells^7^ have been described in detail. In most of the plant cells investigated, actin filaments form a ring structure during prophase. This structure wraps around the nucleus during interphase and participates in the formation of PPB during prophase^8^. During metaphase, the actin filaments are packaged into the spindle and distributed to the cell poles^9^. After cells enter anaphase, actin filaments form the phragmoplast. The phragmoplast expands from the centre of cell plate to both ends. During telophase, the actin filament ring structure is reformed and thin actin filaments are then distributed in the cytoplasm^10^. Actin is considered to be a link in signalling pathways due to its widespread cellular functions, especially in the cell cycle. Plant morphogenesis depends on growth and cell division in the mesophyll. These conditions suggest that actin filaments play important roles in the plant response to ambient light. It is therefore necessary to investigate the role of actin filaments during mitosis in plant leaves.

Although the *in situ* arrangement of the actin cytoskeleton during the cell cycle has already been described, actin arrangement in isolated plant tissues (protoplasts) is not fully understood. A previous study demonstrated that microtubules were depolymerized in wheat protoplasts under enhanced UV-B radiation^11^. However, there has been little research on the roles of actin filaments during mitosis in wheat protoplasts. Some data indicate that dynamic actin controls the de novo induction of polarity in protoplasts^12^. Under abiotic stress, La^3+^ ions can be taken up into plant cells. These ions affect growth via the stabilization of the cytoskeleton in *Zea mays* root protoplasts^13^. In *Zea mays* protodermal root cells, the effects of tungsten were increased; cortical microfilaments were almost completely depolymerized, and intracellular microfilaments appeared highly bundled^14^. Additional research revealed that actin remodelling is involved in NaCl stress tolerance in plants^15^. The leaves are the organs in plants that initially encounter sunlight; therefore, the mitosis process in leaves differs considerably from that in the roots. Hence, the process by which mitosis responds to light signals should be thoroughly understood.

UV-B-induced signalling in plants has been studied; however, the results of this work have been insufficient. Here, we focus on how actin filaments respond to UV-B radiation in wheat protoplasts during the mitosis process. In the present study, wheat protoplasts were isolated from 7-d leaves and then exposed to UV-B radiation. The distribution of actin filaments during the cell cycle was tracked after staining with Ph-FITC. The results further our understanding of the possible mechanisms of UV-B-induced responses in plant leaves and supply evidence of the UV-B signalling pathways in plants.

## Results

### UV-B radiation reduced growth in wheat leaves

**Figure 1:**
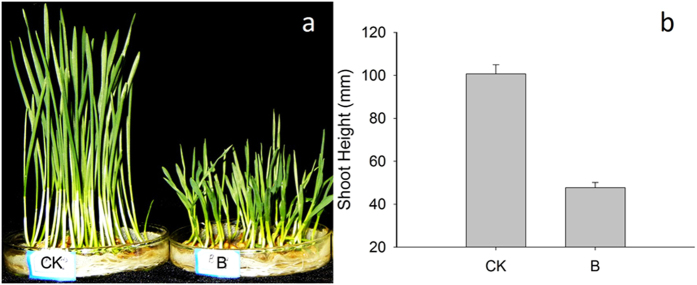
Effects of UV-B radiation on the growth of wheat seedlings. CK: control group. B: UV-B treatment group. Data are means ± SD (n = 3).

The growth of leaves in wheat seedlings under UV-B radiation was significantly inhibited compared to the control group (Fig. 1a). Statistical analysis revealed that wheat shoot height was reduced by 53.12% compared to the control (Fig. 1b). These findings indicate that cell division rates may be responsible for the reduction in leaf growth.

### Isolation of wheat protoplasts and viable activity

**Figure 2:**
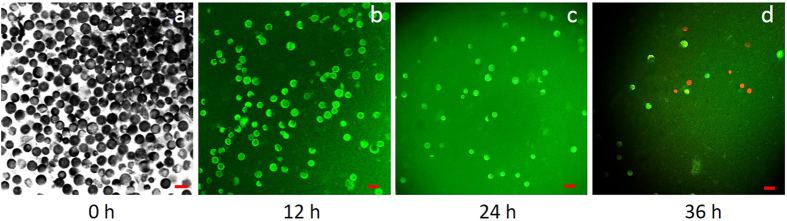
Isolation of mesophyll protoplasts from 7-day-old wheat. (**a**), Image of protoplasts obtained from the confocal DIC channel with a 40× objective. Scale bar = 50 μm. (**b**–**d**), Viable protoplasts were visualized using the FDA staining method; green fluorescing protoplasts were viable (UV-B, 40×). (**b**), 12 h after isolation. (**c**), 24 h after isolation. (**d**), 36 h after isolation. Red protoplasts were considered to be dead. Scale bars = 50 μm.

Seven-day-old wheat leaves were used to prepare protoplasts. Following digestion with enzyme buffer, wheat protoplasts were isolated and detected using a confocal microscope (Fig. 2a). For further study, it is necessary to detect the viability of the isolated protoplasts (Fig. 2b–d). Mesophyll protoplasts were considered viable if green fluorescence was observed. Cells were deemed nonviable if they did not fluoresce or if red fluorescence was observed. The viable activity of protoplasts was measured at three time points (12 h, 24 h, and 36 h). Many green fluorescent protoplasts were observed 12 h after isolation (Fig. 2b), but the total count was less than that at 0 h (Fig. 2a). The total number of protoplasts decreased after 24 h (Fig. 2c), though some red protoplasts were visible at the final time point (Fig. 2d). The viable protoplasts were sufficient for the requirements of the experiment because the subsequent experiment was completed within 12 h.

### Organization of actin filaments during the cell cycle in wheat protoplasts

**Figure 3:**
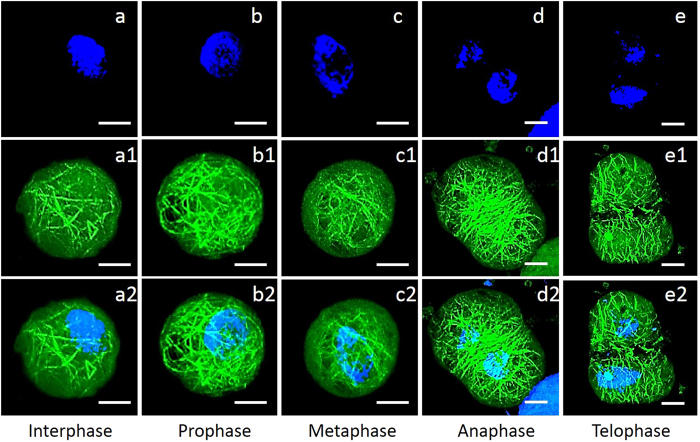
Distribution of actin filaments in wheat protoplasts during mitosis. All of the images shown are superimposed stacks of 15 optical sections. Chromosomes and actin filaments were stained blue with DAPI and green with Ph-FITC, respectively. (**a**–**e**) show the morphology of chromosomes. (**a1**–**e1**) show the arrangement of actin filaments at different phases of mitosis. (**a2**–**e2**) are images a-e merged with (**a1**–**e1**). Actin filaments were dispersed, unordered, and decreased in interphase (**a**–**a2**). The actin filaments polymerized during prophase (**b**–**b2**). During metaphase, actin filaments were found along the equatorial plate (**c**–**c2**). The actin filaments concentrated and polymerized at the phragmoplast and extended to the cell poles in anaphase (**d**–**d2**). After cytokinesis, the distribution of actin filaments was perpendicular to the position of the phragmoplast **(e**–**e2**); the actin filaments spread randomly over time. Scale bars = 10 μm.

Rapid and stable DAPI and Ph-FITC staining allowed for the visualization of the distribution of the nuclei and actin filaments during cell mitosis. During interphase, the actin filaments were found to be dispersed, unordered, and reduced (Fig. 3a1,a2) and the nuclei were holonomic (Fig. 3a). When the cells started to enter mitosis in prophase, large masses of actin filaments were polymerized (Fig. 3b–b2). After the cells entered metaphase, the chromosomes were located on the equatorial plate (Fig. 3c). The distribution of actin filaments was rearranged, and some actin filaments were found on the equatorial plate (Fig. 3c1). In anaphase, during which the two sets of daughter chromosomes separate (Fig. 3d), large masses of long actin filaments gathered between the daughter chromosomes at the phragmoplast. Some actin filaments were extended to the cell poles (Fig. 3d1). After the cells entered telophase, the daughter cell nuclei formed (Fig. 3e). The actin filaments surrounding the phragmoplast disappeared. They were dispersed perpendicular to the phragmoplast (Fig. 3e1) and spread randomly over time.

### Rearrangement of actin filaments in wheat protoplasts under UV-B radiation

**Figure 4:**
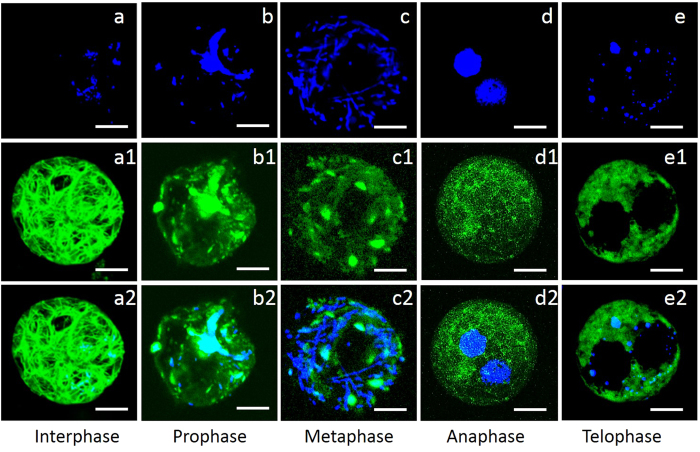
Actin filaments were rearranged in response to UV-B radiation during the cell cycle. All of the images shown are superimposed stacks of 15 optical sections. Actin filaments and nuclei in wheat protoplasts during the cell cycle were stained with Ph-FITC and DAPI, respectively. Actin filaments were bundled during interphase **(a**–**a2**). During prophase, the actin filaments polymerized into patchy structures (**b**–**b2**) and the chromosomes were fragmented. After the protoplasts entered metaphase, actin filaments remained patchy and the chromosomes congressed (**c**–**c2**). Two nuclei appeared in anaphase, and the actin filaments were dispersed without filaments (**d**–**d2**). During telophase, apoptotic bodies emerged. The actin filaments could be stained by Ph-FITC and were distributed in a pool (**e**–**e2**).

To understand how actin filaments change under UV-B radiation, the dynamic processes of the actin cytoskeleton during the cell cycle were observed after treatment with UV-B radiation. During interphase, the actin filaments were bundled as a sturdy rope with high fluorescence intensity (Fig. 4a1); the nucleus was irregular and round (Fig. 4a). After the cells entered mitosis, multiple forms of actin filaments appeared in response to UV-B radiation. During prophase, chromosomes congressed (Fig. 4b); the actin cytoskeleton polymerized into patchy structures without filaments (Fig. 4b1). After the cells entered metaphase, the chromosomes were distributed throughout the cytoplasm (Fig. 4c). The distribution of the actin cytoskeleton was adjacent to the chromosomes and scattered (Fig. 4c1). During anaphase, the actin cytoskeleton was dispersed without filaments (Fig. 4d1) and the protoplast contained two nuclei (Fig. 4d). During telophase, apoptotic bodies emerged in the protoplast (Fig. 4e). The actin cytoskeleton could be stained by Ph-FITC and was distributed in a pool (Fig. 4e1).

### Effects of endogenous drugs on actin filaments in wheat protoplasts

**Figure 5:**
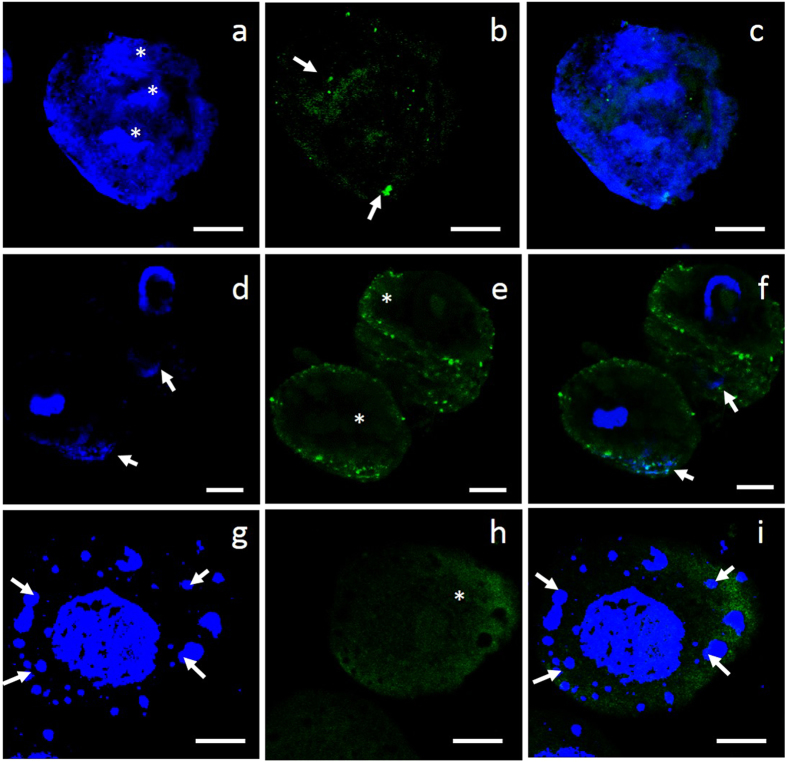
Changes in the organization of the actin cytoskeleton under treatment with CB. All of the images shown are superimposed stacks of 15 optical sections. Chromosomes and actin filaments were stained blue with DAPI and green with Ph-FITC, respectively. (**a**,**d**,**g**) show nuclei; (**b**,**e**,**h**) show actin filaments. Figures (**c**,**f**,**i**) are merged images. Actin filaments were fully depolymerized after treatment with CB (**b**,**e**,**h**), as indicated by the white arrowheads in Fig. e and the white asterisks in Figs (**e**,**h**). The morphology of the nuclei changed into three bundles (white asterisks in Fig. a), chromosomes lagged (white arrowheads in Figs (**d**,**f**)), and apoptotic bodies were visible (white arrowheads in Figs (**g**,**i**)).

To investigate whether actin filaments in wheat protoplasts tend to depolymerize or polymerize in response to UV-B radiation, pharmacological experiments using the special drugs CB and JAS were performed. Actin filaments were depolymerized into small debris after treatment with CB (Fig. 5b,e,h). The nuclei were abnormal and appeared as three bundles (Fig. 5a). Chromosomes lagged (Fig. 5d), and apoptotic bodies were visible (Fig. 5g).

**Figure 6:**
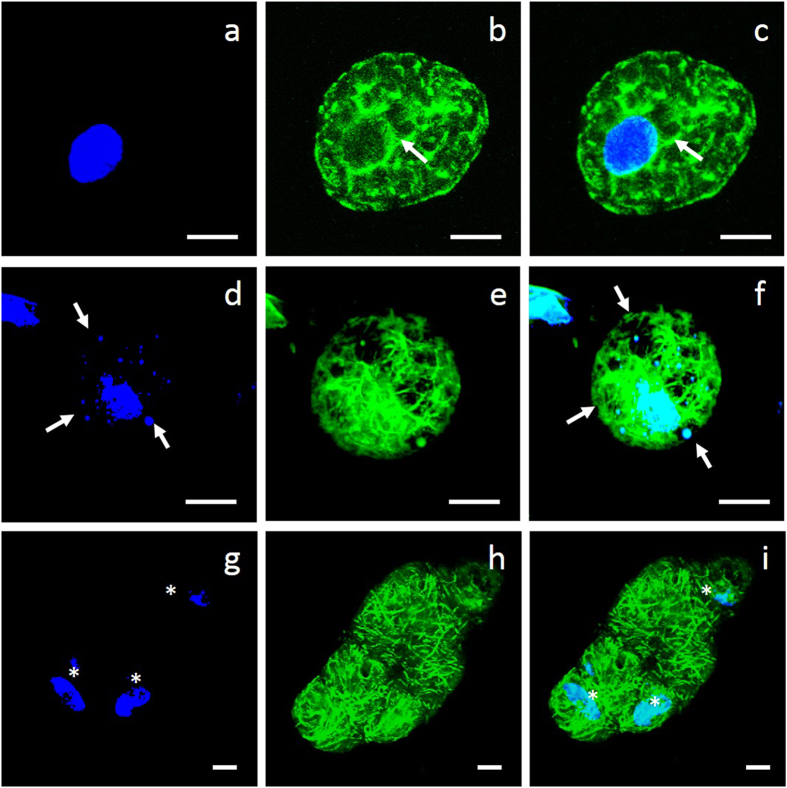
Actin filaments were bundled in response to endogenous JAS. All of the images shown are superimposed stacks of 15 optical sections. Chromosomes and actin filaments are shown in blue and green, respectively. Each row of images shows a single cell, and each column represents a fluorescence channel. Actin filaments polymerized into thick bundles as shown in Figs (**b**,**c**) (white arrowheads). However, apoptotic bodies appeared with the actin bundles (white arrowheads in Figs (**d**,**f**)). In some cases, the wheat protoplasts fused together as shown in the last row (Figs (**g**–**i**)); the white asterisks indicate three nuclei.

High levels of fluorescence were observed in the protoplasts due to the polymerization of actin filaments into thick bundles (Fig. 6b,e) accompanied by apoptotic bodies (Fig. 6d). In some cases, the wheat protoplasts fused together with large masses of actin filaments and multiple nuclei (Fig. 6g–i).

## Discussion

Our understanding of the mechanisms of the cell cycle continues to improve, especially in plant cells. Many studies of mitosis have performed in model plants, such as tobacco BY-2 cells^16^ and *Arabidopsis*^17^. However, fewer studies have been carried out in wheat cells such as wheat protoplasts. Previous research demonstrated that actin filaments participated in the process of “partition bundle division” in wheat root cells under enhanced UV-B radiation^7^. In contrast to the roots, wheat leaves are the organs that first capture UV-B radiation. It is important to research the response of actin filaments exposed to UV-B radiation in wheat protoplasts.

Here, actin filaments were clearly redistributed under UV-B radiation. During interphase, dispersed, unordered actin filaments polymerized into thick bundles in response to UV-B radiation; actin filaments changed into patches during prophase and metaphase. When the cells entered into anaphase and telophase, actin filaments depolymerized and dispersed without filamentous. After protoplasts were incubated with specific drugs (CB and JAS), actin filaments fragmented or stabilized into thick bundles. Regardless of the treatment applied, cell death occurred. Under UV-B radiation, the actin filaments were rearranged and exhibited different phenotypes at every stage during the cell cycle. These results indicated that the mechanism by which actin filaments respond to UV-B radiation is very complicated and cannot be understood based on a single representation.

Although the mechanism by which actin filaments respond to UV-B radiation has not been fully described, changes to actin filaments in response other stressors have been reported. Under low temperature, phragmoplast actin filaments were chaotic; the normal cell plate failed to form in a thermo-sensitive male sterile line of wheat^18^. Treatment with 200 mM NaCl disrupted actin filament dynamics within 10 min and increased the ROS levels in the elongation zone cells of *Arabidopsis* root tips^15^. Biochemical experiments revealed that pH plays a key role in the polymerization and depolymerization of actin^19^. A recent study found that UV-B radiation induced cytosolic alkalization in the guard cells of *Arabidopsis*^20^. Taken together, these studies demonstrated that actin could be an important functional protein in the plant response to environmental factors. Many actin-binding proteins and actin-related proteins participate in the process of rearranging actin filaments. However, the regulatory mechanism should be investigated.

**Figure 7:**
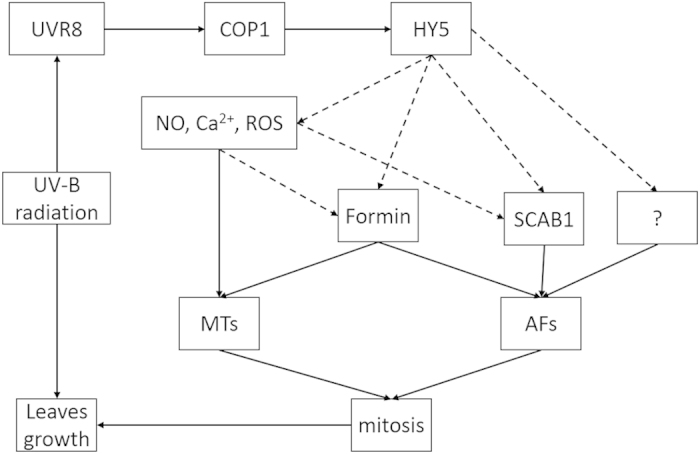
Schematic of the possible mechanisms of actin filaments in response to UV-B radiation. Solid lines indicate confirmed results, and dotted lines show suppositional processes. The question mark represents unknown proteins. UVR8: UV Resistance Locus 8. COP1: Constitutively Photomorphogenic 1. HY5: Elongated Hypocotyl 5. ROS: Reactive Oxygen Species. SCAB1: Stomatal Closure Related Actin Binding Protein 1. MTs: Microtubules. AFs: Actin Filaments.

Recently, researchers have paid increasing attention to UV-B signalling pathways that are mediated through the UVR8 protein. UVR8 has been shown to be the target receptor of UV-B radiation in plants^21^. Absorption of UV-B radiation dissociates the UVR8 dimer into monomers, initiating signal transduction through interactions with COP1 and HY5^5^. The expression of a large number of genes is influenced by HY5^22^; however, the downstream signalling molecules in UVR8 signalling pathways remain unclear. Here, our results show that the distribution of actin filaments is altered during cell mitosis in response to UV-B signals. A previous study revealed that UV-B radiation reduced the rate of cell division^23^, which is dependent on the cytoskeleton. The organization of microtubules (randomized, fragmented or depolymerized), as one component of the cytoskeleton, under UV-B radiation has been investigated in *Arabidopsis*^24^. Few reports have focused on the response to UV-B radiation in other cytoskeleton components, such as the rearrangement of actin filaments during cell mitosis. A report demonstrated that cytoskeletal proteins are molecular targets for NO signals in plants^25^. As a linking regulator, the rice formin protein formin homology 5 (FH5) plays a critical role in determining plant morphology by regulating the dynamics of actin and the proper spatial organization of microtubules and microfilaments^26^. This finding implies that formin proteins act as upstream factors of actin filaments and microtubules and downstream factors of HY5 and NO^27^. There may also exist unknown factors that receive signals from UVR8/COP1/HY5 and regulate actin filaments, which may influence the rate of cell mitosis and seedling growth (Fig. 7).

## Conclusion

Our results clearly reveal the dynamic organization of the actin cytoskeleton during mitosis and cytokinesis in wheat protoplasts after treatment with UV-B radiation. These findings indicate that actin filaments respond to UV-B radiation though rearrangement and influence the mitosis process in plant protoplasts, ultimately inducing shorter leaves under UV-B radiation.

## Materials and Methods

Linyou 2871 (*Triticum aestivum* L. cv.) kernels were supplied by the Wheat Research Institute of Agricultural Sciences in Shanxi Province, China. Wheat seeds were selected and sterilized for 10–15 min with 1% NaClO. The seeds were then washed for 10 min with running water. Thirty seeds per Petri dish were cultured on wet filter paper in a growth chamber at 25 °C and 60% relative humidity and watered daily. UV-B radiation was applied for 8 h/day during the light cycle (8 h light and 16 h dark), and the intensity was 10.08 KJ/m^2^/d^28^. After seven days of growth, fully expanded leaves were excised and prepared for the isolation of the protoplasts. All of the experiments were performed with three replications.

### Isolation of protoplasts

Seven-day-old wheat leaves were cut into 1-mm segments and treated separately in different Petri dishes with an enzyme solution containing 0.25% macerozyme R 10 and 1% cellulase R 10 (Yakult Co., Japan) in W5 salt solution (154 mM NaCl, 125 mM CaCl_2_, 5 mM KCl, 2 mM MES-KOH, pH 5.7). Excised tissue was incubated for 3.5 h in a thermostat at 25 °C without shaking. Incubated mixtures were filtered separately through a 50-μm nylon mesh and transferred to 10-mL centrifuge tubes. Suspensions were centrifuged at 500 rpm for 5 min. After the supernatant was carefully removed, 4 mL of 20% sucrose solution was mixed with the protoplast suspension. The mixture was gently covered with 2 mL W5 salt solution without disturbing the protoplast suspension. Suspensions were again centrifuged at 500 rpm for 5 min. Floating protoplasts, forming a ring between the layers of W5 and sucrose solutions, were collected using a sterile pipette. The protoplasts were diluted in W5 solution and centrifuged at 500 rpm for 2 min^29^,^30^.

### Protoplast viability determination

Fluorescein diacetate (FDA; Sigma Co., USA) was stored in an acetone stock solution (5 mg/mL) at 4 °C. FDA was added to the protoplast suspension to a final concentration of 0.01%. After 5 min at room temperature, the protoplasts were examined for fluorescence using an Olympus fluorescent microscope (with an excitation wavelength of 488 nm and an emission wavelength of 530 nm)^31^.

### UV-B and exogenous drug treatments

UV-B radiation was generated using a UV-B lamp lain on the top of the protoplasts suspensions. The dose of the UV-B radiation was 10.08 KJ/m^2^/d^28^; suspensions were irradiated for 4 h. Exogenous drugs were added to the protoplasts suspensions^32^; the final concentration of CB was 2.0 μg/mL, and the final concentration of JAS was 30 nmol/L.

### Fluorescence imaging

Cellular fluorescence was monitored using an FV-1000 Confocal system (Olympus Co., Japan) with a 1.4 NA, 60× oil immersion objective lens. Cells from at least three replicate samples were observed. Double fluorescent labelling with 4’,6-diamidino-2-phenylindole DAPI (Sigma, USA) and Ph-FITC (Cytoskeleton Co., USA) was carried out in wheat protoplasts^6^. Incubations with FITC-Ph (50 μg/mL) were performed at 37 °C for 40 min. Incubations with DAPI (100 ng/mL) were performed at 37 °C for 10 min. Actin filaments were stained with Ph-FITC, and an excitation wavelength of at 488 nm was applied. Nuclei were stained with DAPI, and an excitation wavelength of 405 nm was applied. The images collected were 512 × 512 pixels in size.

### Statistical analysis

Data analysis was performed using IBM SPSS Statistics (version 21.0). The least significant difference test at a 5% probability level was applied to test the differences among the mean values of each attribute.

## Additional Information

**How to cite this article**: Chen, H. and Han, R. Characterization of Actin Filament Dynamics during Mitosis in Wheat Protoplasts under UV-B Radiation. *Sci. Rep.* **6**, 20115; doi: 10.1038/srep20115 (2016).
